# Allelopathy and its coevolutionary implications between native and non‐native neighbors of invasive *Cynara cardunculus* L.

**DOI:** 10.1002/ece3.6472

**Published:** 2020-06-28

**Authors:** Md. Nazim Uddin, Takashi Asaeda, Shahana H. Shampa, Randall W. Robinson

**Affiliations:** ^1^ Institute for Sustainable Industries and Liveable Cities College of Engineering and Science Victoria University Melbourne Vic. Australia; ^2^ Department of Environmental Science Saitama University Saitama Japan; ^3^ Institute for Studies of the Global Environment Sophia University Chiyoda Tokyo Japan

**Keywords:** allelopathy, co‐occurring plants, *Cyanara cardunculus* L., evolved tolerance, invasive plant, plant invasion, rhizosphere

## Abstract

Invasive plants apply new selection pressures on neighbor plant species by different means including allelopathy. Recent evidence shows allelopathy functions as remarkably influential mediator for invaders to be successful in their invaded range. However, few studies have determined whether native and non‐native species co‐occurring with invaders have evolved tolerance to allelopathy. In this study, we conducted germination and growth experiments to evaluate whether co‐occurring native *Juncus pallidus* and non‐native *Lolium rigidum* species may evolve tolerance to the allelochemicals induced by *Cyanara cardunculus* in Australian agricultural fields. The test species were germinated and grown in pots filled with collected invaded and uninvaded rhizosphere soil of *C. cardunculus* with and without activated carbon (AC). Additionally, a separate experiment was done to differentiate the direct effects of AC on the test species. The soil properties showed invaded rhizosphere soils had higher total phenolic and lower pH compared with uninvaded soils. We found significant reduction of germination percentage and seedling growth in terms of above‐ and belowground biomass, and maximum plant height and root length of native in the invaded rhizosphere soil of *C. cardunculus*, but little effect on non‐native grass species. Even soil manipulated with AC showed no significant differences in the measured parameters of non‐native except aboveground biomass. Taken together, the results indicate allelochemicals induced by *C. cardunculus* exert more suppressive effects on native than non‐native linking the coevolved tolerance of those.

## Graphical abstract

Significant allelopathic effects of rhizosphere soil in *Cynara cardunculus* have been observed in terms of germination and growth reduction of associated native, but less effect on non‐native grass species. The results indicate the co‐evolved tolerance of native and non‐native in the introduced range, where native species showed severe sensitivity, but non‐native was more resistance.

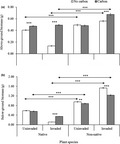

## 1 INTRODUCTION

Biological invasion plays fundamental role for ecosystem dysfunctions through loss of biodiversity across the world (Cordero, Torchelsen, Overbeck, & Anand, 2016; Heringer, Thiele, Meira‐Neto, & Neri, 2019; Mack et al., 2000). Thus, it prompts wide research to unravel invasion mechanisms and consequences of invasive species into natural systems. Therefore, this advances our understanding of mechanisms and effects of those invaders that play an important role in altering community structure and ecosystem functions (Castro‐Diez, Pauchard, Traveset, & Vila, 2016; Lodge, 1993; Sodhi, Livingstone, Carboni, & Cadotte, 2019). There are many hypotheses may explain the success of the invasive species in the introduced range (Catford, Jansson, & Nilsson, 2009; Crandall & Knight, 2018). Specifically, “enemy release”—lack of coevolutionary history with its host in the introduced range (Allen et al., 2017); “novel weapons”—gaining a competitive advantage over native by possessing novel biochemical (Becerra et al., 2018); and “evolution of increased competitive ability”—evolving more competitive ability by releasing most specialist enemies following introduction (Montesinos, Graebner, & Callaway, 2019) are commonly used. Biochemical interactions among plants have revived interest in invasion ecology (Callaway & Maron, 2006), which is referred as “allelopathy.” The “allelopathy” was defined by Rice (1984): “any direct or indirect harmful or beneficial effect by one plant (including microorganisms) on another through production of chemical compounds that escape into the environment.” However, it has been acknowledged with doubt for decades due to its complexity in demonstration (Keeley, 1988; Meiners, Kong, Ladwig, Pisula, & Lang, 2012; da Silva, Overbeck, & Soares, 2017). However, contemporary research provides evidence of allelopathy between invasive and native plant species, which might be one of the underlying mechanisms of invaders’ success into invasion processes (Bais, Vepachedu, Gilroy, Callaway, & Vivanco, 2003; Callaway & Ridenour, 2004; Ooka & Owens, 2018; Prati & Bossdorf, 2004; Song, Qin, He, Wang, & Yu, 2019; Stinson et al., 2006; Uddin, Robinson, Buultjens, Al Harun, & Shampa, 2017). Additionally, some studies found success of the invader is in part due to the introduction of novel allelopathic compounds, as many co‐occurring plant species exhibit a lack of evolved tolerance to these compounds (Callaway & Ridenour, 2004; Huang, Lankau, & Peng, 2018; Lankau, 2012; Lyytinen & Lindstrom, 2019).

The debate on allelopathy in natural ecosystems is partially characterized by methodological difficulties to demonstrate the effects of the natural concentration level of allelochemicals on coexisting plant species (Mallik, 2008). A majority of allelopathic studies have been conducted using plant extracts from different solvents and plant materials under laboratory conditions which are ecologically unrealistic (Inderjit & Dakshini, 1995) (Hierro & Callaway, 2003; Inderjit & Callaway, 2003; Inderjit & Weston, 2000). In spite of advancement in using ecologically “realistic” technique, experiments evaluating the evolved tolerance of neighboring plant species (native versus non‐native) to root exudates found in rhizosphere are exceptionally rare (Inderjit & Nilsen, 2003; Lankau, 2012; Lyytinen & Lindstrom, 2019), with a few exceptions (Blair, Weston, Nissen, Brunk, & Hufbauer, 2009; Fujii et al., 2004; Nilsson, 1994; Nilsson, Zackrisson, Sterner, & Wallstedt, 2000). Noteworthy here is that the study regarding *C. cardunculus* by Scavo, Rial, Molinillo, et al. () has been carried out to improve the methodological aspects of allelopathy. The experiments in the field and/or using field level allelochemical concentration in rhizosphere with co‐occurring plant species and demonstrating the evolved tolerance of those may offer the possibility to overcome the debate on allelopathic effects (da Silva et al., 2017; C. Fernandez et al., 2016; Jose & Gillespie, 1998; Scavo, Abbate, & Mauromicale, 2019; Zackrisson & Nilsson, 1992).

*Cynara cardunculus*, native to the Mediterranean basin, is a herbaceous perennial thistle (Pignone & Sonnante, 2004) and has invaded in the western USA, South America, UK, Australia, and New Zealand (GISD, 2020). In Australia, *Cynara cardunculus* subsp. *flavescens* Wiklund is declared as a significant environmental weed in the states of Victoria, South Australia, and Tasmania (Bean, 2017), and the variety is unclear but it might be *scolymus* (L.) Benth (Bean, 2015). *C. cardunculus* is considered a pest in agriculture systems, including pasture land and disturbed areas, and it invades natural habitats including grasslands, riparian areas, open woodlands, and wetlands.

Though *C. cardunculus* has higher vegetative reproduction from its roots and crowns of the mature plant, it also produces higher seeds ranged from 600 to 30,000 seeds/year/plant (Marushia & Holt, 2006, 2008). It is very fast growing with higher above and belowground biomass, and more competitive than associated native plant species. These attributes could contribute to the invasiveness of this plant (White & Holt, 2005). The widespread distribution and dense monocultures (20,000 plants per acre) displace native plant communities and reduce plant diversity across the world (Marushia & Holt, 2006; White & Holt, 2005). The dense populations of *C. cardunculus* also cause the restriction of wildlife movement and native vegetation growth creating fragmentation of native habitats (Kelly & Pepper, 1996). For instance, the invasion of *C. cardunculus* displaced the endangered plant species *Acanthomintha ilicifolia* in California (Kelly, 2000).

With these common invasive traits, the allelopathic trait of *C. cardunculus* has contributed to the displacement and reduction of native biodiversity (Carlos Rial, Novaes, Varela, Molinillo, & Macias, 2014; Scavo, Pandino, et al., 2019; Scavo, Restuccia, Abbate, & Mauromicale, 2019; Scavo, Restuccia, Pandino, Onofri, & Mauromicale, 2018). It produces a variety of allelochemicals including phenolic compounds (Pandino, Lombardo, Mauromicale, & Williamson, 2011a, 2011b), sesquiterpene lactone (Rial et al., 2016; Scavo, Rial, Molinillo, et al., ), and polyphenols (caffeoylquinic acids and flavonoid derivatives) (Ciancolini, Alignan, Pagnotta, Vilarem, & Crino, 2013). Those allelochemicals demonstrated inhibitory effects on wheat coleoptile, standard target species, and weed growth (Rial et al., 2014, 2016). These compounds may be available through biomass leaching and decomposition, and root exudates that makes substantial concentration in the rhizosphere soil to influence seed bank of associated plant species (Scavo, Restuccia, et al., 2019).

Most of the past studies into *C. cardunculus* have focused on medicine (Bras, Guerreiro, Duarte, & Neves, 2015; Garbetta et al., 2014; Koubaa, Damak, McKillop, & Simmonds, 1999; Miadokova et al., 2008; Yasukawa, Matsubara, & Sano, 2010), food (Agboola, Chan, Zhao, & Rehman, 2009; Dias et al., 2018; Pandino, Lombardo, Mauromicale, & Williamson, 2011a, 2011b), and biofuel (Ciancolini et al., 2013; J. Fernandez, Curt, & Aguado, 2006; Gominho, Curt, Lourenco, Fernandez, & Pereira, 2018). Some studies have tested allelopathy using plant extract in its native range (Carlos Rial et al., 2014; Scavo, Pandino, et al., 2019; Scavo, Restuccia, et al., 2019; Scavo et al., 2018), and competitive ability of *C. cardunculus* (White & Holt, 2005). While those studies have demonstrated allelopathic effects of *C. cardunculus* on model and weed species in its native range, none has examined the effects on associated native and non‐native grass species. More specifically, no studies evaluate the evolved tolerance of the co‐occurring native and non‐native grass species with field concentrations of root exudates (allelochemicals) available in rhizosphere of *C. cardunculus*. To the best of our knowledge, to date no allelopathic studies have been conducted in its non‐native range, specifically in Australian *C. cardunculus* populations.

## 2 MATERIALS AND METHODS

Our experiments were set to test the allelopathic effects of *C. cardunculus* on germination and growth of grass species that co‐occurs in agriculture field in Victoria, Australia. We used field‐collected rhizosphere soils to imitate natural conditions through exposing of target species that minimize uncertainties in plant extract concentrations or exclusion of other possible edaphic effects (Gómez‐Aparicio & Canham, 2008). As the allelopathic effects are species‐specific (Callaway & Ridenour, 2004; Gomez‐Aparicio & Canham, 2008), we also devised the experiments to determine the evolved tolerance of associated native versus non‐native grass species to the allelopathic potential of *C. cardunculus* in its invaded community. In addition, chemical properties (total phenolic content and pH) of *C. cardunculus* rhizosphere were assessed to test the differences among the populations. Overall, this study aimed to evaluate allelopathy and coevolutionary effects of rhizosphere soil induced by *C. cardunculus* on co‐occurring native and non‐native grass species in its invaded range.

### 2.1 Measurement of field soil chemical properties

To test whether invasive plant species *C. cardunculus* changes chemical soil properties, we collected rhizosphere soil at the end of November 2018 during its highest biomass production from three invaded and three nearby noninvaded populations in agricultural land at 26.7 m above sea level in Werribee South, Victoria, Australia (37°54′44.5″S 144°42′00.8″E). We designed our sampling protocol to minimize the effects through selection of homogenous soil profile, and plant density and diversity in uninvaded and invaded sites of each population, which were also close to each other. The location of each population including soil from both invaded and nearby noninvaded was separated from the others by a distance of at least 500 m. At each location, we collected five samples from five individuals of *C. cardunculus* as invaded soil samples and five from nearby noninvaded population where other species such as *Juncus pallidus*, *Maireana decalvans*, and *Themeda triandra*except *C. cardunculus* were present. After that, the soil samples were transported into the laboratory, sorted and homogenized for composite sample. Then, we took five subsamples from each population for measurement of soil properties and measured total phenolic content (TPC) and pH in soil as it is assumed those properties might play a significant role in testing allelopathy, though rhizosphere has also numerous aspects (Scavo, Abbate, et al., 2019). Soil pH was determined with a pH meter (Pocket digital pH meter, 99,559, Dick Smith Electronics, Australia) in a 1:2.5 w/v (soil: distilled water) ratio (Paz‐Ferreiro, Trasar‐Cepeda, Leirós, Seoane, & Gil‐Sotres, 2007). Soil phenolics was determined by sampling 100 mg of air‐dried soil following the Folin–Ciocalteu method (Blainski, Lopes, & De Mello, 2013).

### 2.2 Germination experiment

However, the multiple populations provide the possibility to check the variation among populations through increasing the reliability of the results, and better representing the condition of each treatment, but we avoided using multiple populations in this occasion due to complexity in research design and the possibility of confusion. The collected homogenized rhizosphere soil from only one population was used in the experiment as no attempt was made to check variation among populations. Half of the soil (invaded and noninvaded) was thoroughly mixed with activated carbon (AC) and the mixture was placed into the pots as a treatment. The remaining soil was considered as a treatment without AC. The AC has special characteristics with high absorptivity of organic complexes, such as allelochemicals, and weak affinity for inorganic molecules, such as those present in nutrient solution. Many allelopathic studies related to *C. stoebe* including *C. maculosa*, *C. rhenana, C. muretii, C. vallesiaca,* and *C. stoebe* subsp. have demonstrated through reducing the suppressive effects of root exudates (Callaway, Ridenour, Laboski, Weir, & Vivanco, 2005; Lyytinen & Lindstrom, 2019; Ridenour & Callaway, 2001). The 100 g soils were placed in punnets (9.75 cm by 6.75 cm) with five replicates. The punnets were moistened and kept at room temperature for 24 hr to activate the soil microbes. The sterilized seeds (collected from fields invaded by *C. cardunculus*) of native *Juncus pallidus* and non‐native *Lolium rigidum* grass species were then seeded at a rate of 20/pot. The tested seeds were selected due to the following reasons: (a) They are co‐occurring plant species of *C. cardunculus*; (b) allelopathic interactions between invasive alien and coexisting plant species (native and non‐native) seem to be one of the underlying mechanisms for the invasion success of some invaders; and (c) testing the evolved tolerance of the coexisting species. We followed a complete randomized block design in this study. The pots were kept in shade for 3 days and then transferred to the natural lit greenhouse at 23 ± 3°C day and 12 ± 2°C night temperatures. The pots were watered with automated irrigation system twice in a day. Pots were randomly shuffled every week to minimize the spatial effects. Then, after four weeks, the germinant was counted for each treatment.

### 2.3 Growth and establishment experiment

The soils were prepared as per the abovementioned germination experiment, and the 100g soils were placed in punnets (9.75 cm by 6.75 cm) for growth experiment. However, the size of the punnets may seem tighten for growth of whole period of the experiment, but the plants can adopt with it, and it may not interfere the findings as it was applicable for all replicates of both tested species. About 300 (three hundred) sterilized seeds of each grass species (native and non‐native) were germinated in a tray (30 cm by 28 cm) filled with sterilized sand. One seedling of both species was then transplanted into the prepared punnet by twenty‐one replicates and pots were grouped by target species following a randomization across treatments and kept as per the abovementioned condition. Dead seedlings were replaced after a period of two weeks, to allow the experiment to continue. The pots were watered using an automated irrigation system twice in a day and fertilized fortnightly with liquid fertilizer (Hyponex, N‐P‐K, 6‐10‐5, Hyponex Inc) at a concentration of 2ml/1 of water. Pots were randomly shuffled every week to minimize the spatial effects, and after 3.5 months, the plants were harvested for biometric parameters including maximum plant height and root length, aboveground biomass (AGB), belowground biomass (BGB), total biomass (AGB plus BGB), and AGB‐BGB ratio.

We conducted a separate experiment along with the main experiments to assess the direct effect of AC on seedlings growth of native and non‐native used grass species with sterilized sand and AC treatments. We used five replicates per treatment (with and without AC) for both of grass species. The direct effect of AC on plant growth has been assessed due to its complication on allelopathy (Lau et al., 2008). This experiment was carried out adopting the same procedure as the aforementioned experiment and measured biometric parameters (Appendix S1).

### 2.4 Data analyses

An independent sample *t* test was performed to check the differences of soil phenolics and pH level among uninvaded and invaded sites for each population of *C. cardunculus*. Two‐way ANOVAs were also conducted to test the differences of those variables as functions of populations and invasion status (uninvaded and invaded). We again conducted two‐way ANOVAs to test the effect of soil allelopathy as functions of activated carbon (with and without AC) and invasion status (uninvaded and invaded) on AGB, BGB, AGB‐BGB ratio, total biomass, and maximum plant height and root length of native and non‐native grass species. Target species (native and non‐native) were not considered as third fixed factor in the univariate general linear model due to avoid complexity. For example, the test does not provide which specific species is significantly different from each other. As a result, separate analysis (species‐specific) was done to get direct understanding of the allelopathic effects on each of them. Moreover, an independent sample *t* test was performed to compare the significant differences between AC treatments (with vs. without carbon) and species (native vs. non‐native) in both of germination and growth experiments including separate growth experiment. To maintain homogeneity of variances and normality of the used data for these analyses, we used data transformation techniques: a square root (soil phenolics, AGB, BGB, total biomass for native; AGB for non‐native) and an arcsine‐square root function (germination percentage for native). We also applied Levene's test for homogeneity of variance and Kolmogorov‐Smirnov (K‐S) test for normality of the used data. All data were analyzed using SPSS version 20.0 (IBM Corporation).

## 3 RESULTS

### 3.1 Rhizosphere soil chemical properties

**FIGURE 1:**
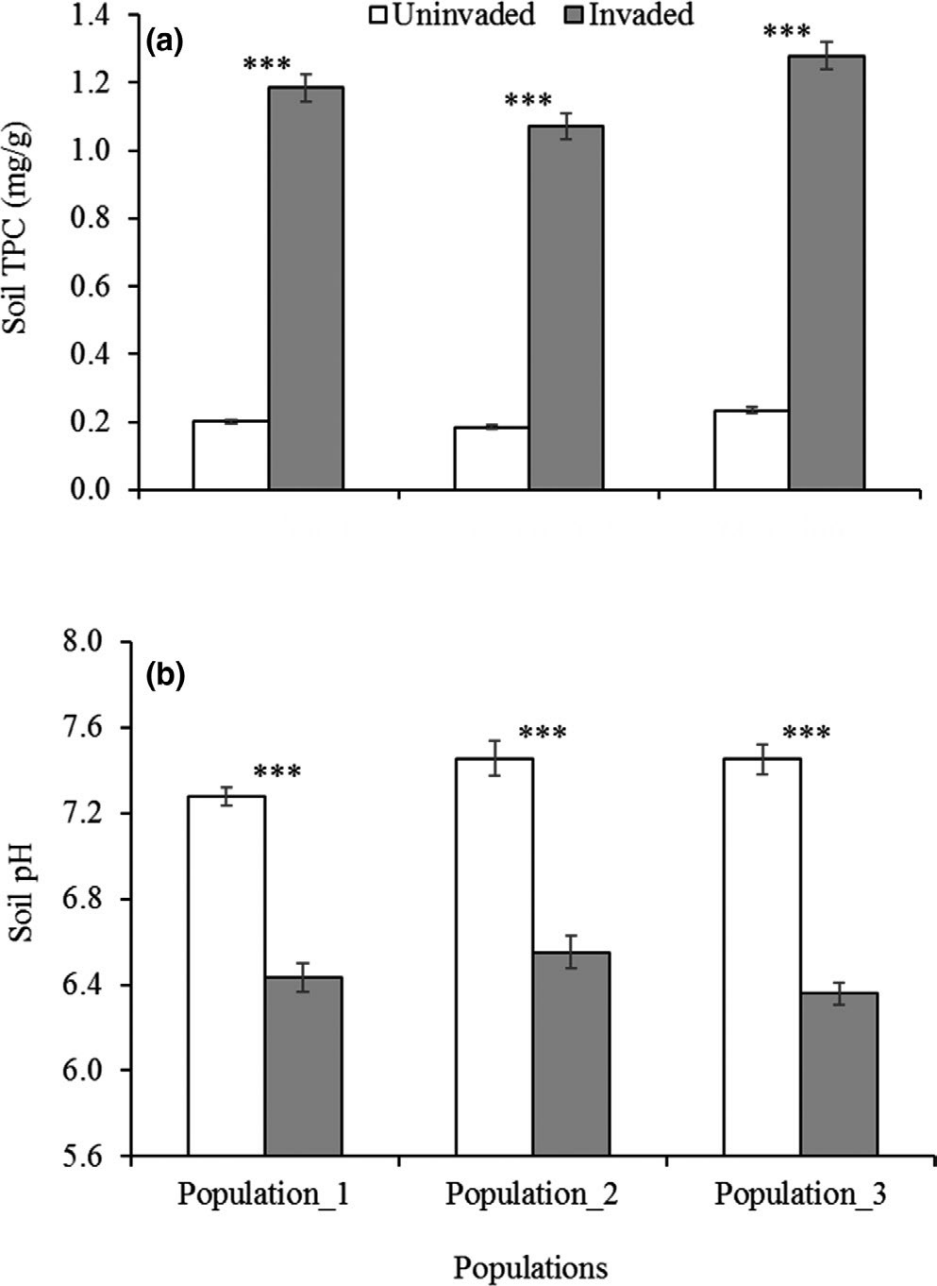
Changes to (a) phenolics and (b) pH due to *Cynara cardunculus* invasion in three populations. Each bar is the mean ± *SE*, *n = 5*. Asterisks designate significant difference between uninvaded and invaded plots within each population after independent sample *t* test (***p ≤ .001)

The measured soil chemical properties such as total phenolics and pH have been altered due to *C. cardunculus* invasion. The soil phenolics and pH of all populations was significantly higher and lower, respectively, at invaded sites compared to uninvaded sites (phenolics: *F*_1,24_ = 3,291.77, *p* ≤ .001; pH: *F*_1,24_ = 304.06, *p* ≤ .001) (Figure 1). The significant variation of only phenolics among populations was observed (*F*_2,24_ = 17.47, *p* ≤ .001). However, there were no interactions between populations × invasion status on those variables (phenolics: *F*_2,24_ = 1.18, *p* = .32; pH: *F*_2,24_ = 1.45, *p* = .25). The phenolic content varied with a range of 0.18 to 0.23 mg/g at uninvaded and 1.07 to 1.28 mg/g at invaded sites among populations, whereas the pH level was 7.27 to 7.45 at uninvaded and 6.35 to 6.55 at invaded sites.

### 3.2 Germination test

**FIGURE 2:**
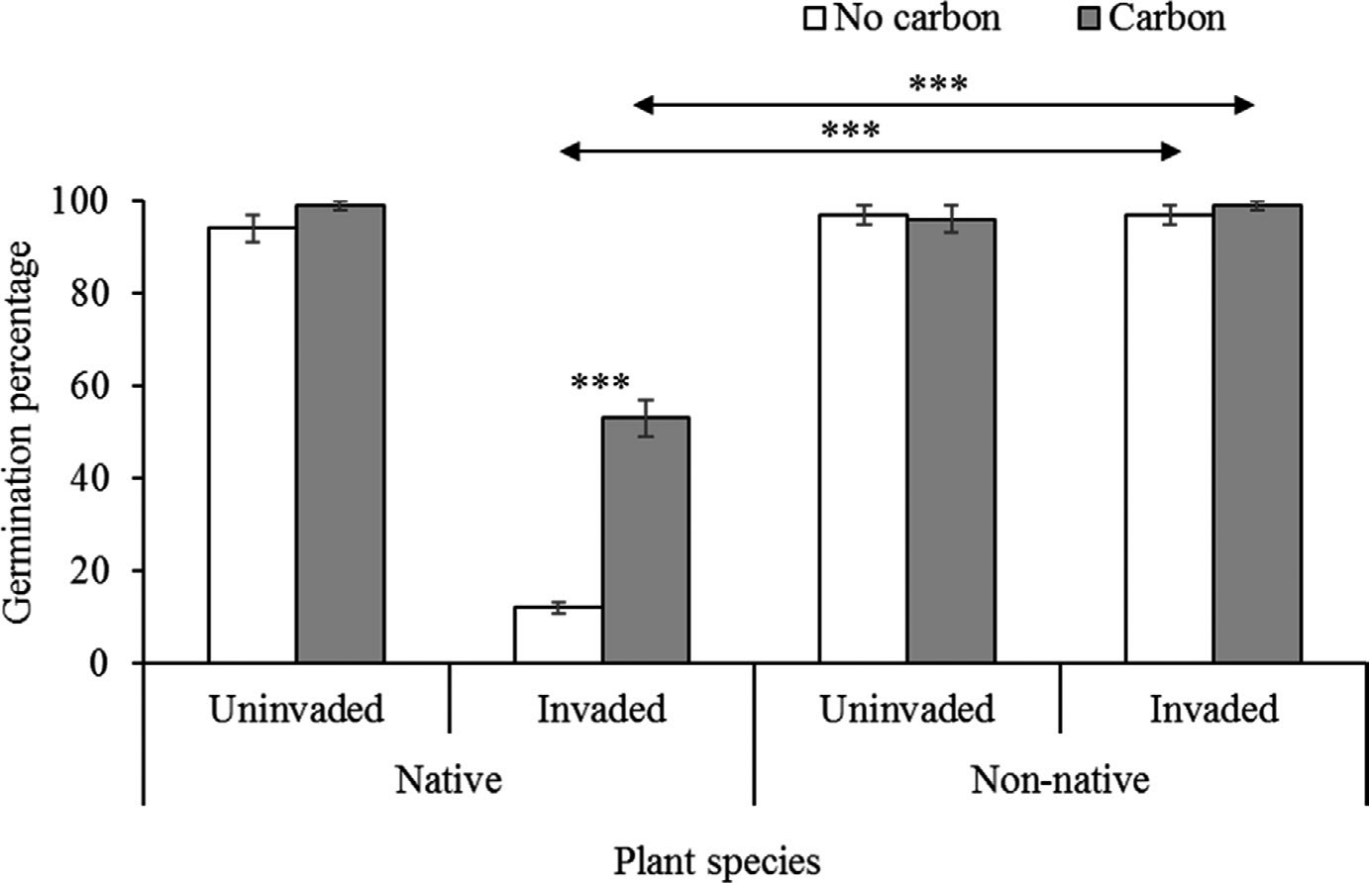
Germination percentage of native *Juncus pallidus* and non‐native *Lolium rigidum* grass species germinated in uninvaded and invaded rhizosphere soil of *Cynara cardunculus* either with or without activated carbon (AC). Each bar is the mean ± *SE*, *n = 5*. Asterisks above bar and horizontal line designate significant difference between AC treatments and target species, respectively after independent sample *t* test (***p ≤ .001)

There was significant interactive effect of invasion status and carbon treatments on germination percentage of the native species (*F*_1,16_ = 26.50, *p* ≤ .0001), but no significant effect on non‐native species (*F*_1,16_ = 0.51, *p* = .48) (Figure 2). The germination percentage of native species was also influenced significantly by the main effect of invasion status (*F*_1, 16_ = 360.29, *p* ≤ .0001) and AC treatments (*F*_1, 16_ = 59.24, *p* ≤ .0001), whereas non‐native species was neither affected by invasion status (*F*_1, 16_ = 0.51, *p* = .48) nor AC treatments (*F*_1, 16_ = 0.06, *p* = .81). The AC in invaded rhizosphere soil increased 341% germination for native. However, it was only 2.1% for non‐native grass seeds indicating that soil allelopathy of *C. cardunculus* had strong negative effect on native compared to non‐native seed germination (Figure 2). In addition, there was significant difference between native and non‐native species in without (*t* = 36.24, *df* = 8, *p* ≤ .001) and with (*t* = 11.63, *df* = 8, *p* ≤ .001) AC treatments in invaded soil, but no effect in uninvaded soil (Figure 2).

### 3.3 Seedling growth performance

**FIGURE 3:**
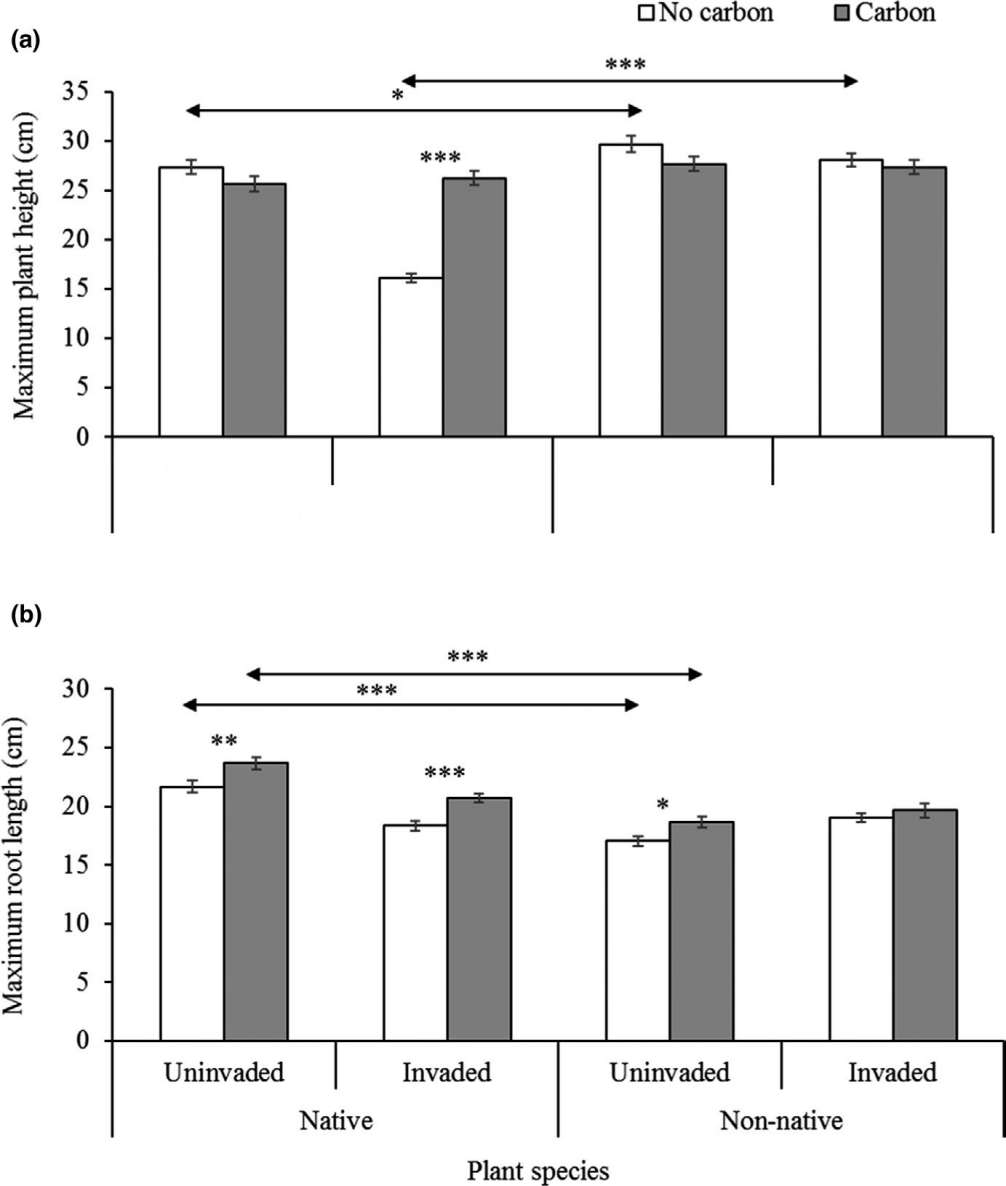
(a) Maximum plant height and (b) maximum root length of native *Juncus pallidus* and non‐native *Lolium rigidum* grass species grown in uninvaded and invaded rhizosphere soil of *Cynara cardunculus* either with or without activated carbon (AC). Each bar is the mean ± *SE*, *n = 21*. Asterisks above bar and horizontal line designate significant difference between AC treatments and target species, respectively after independent sample *t* test (*p ≤ .05; **p ≤ .01 & ***p ≤ .001)

**FIGURE 4:**
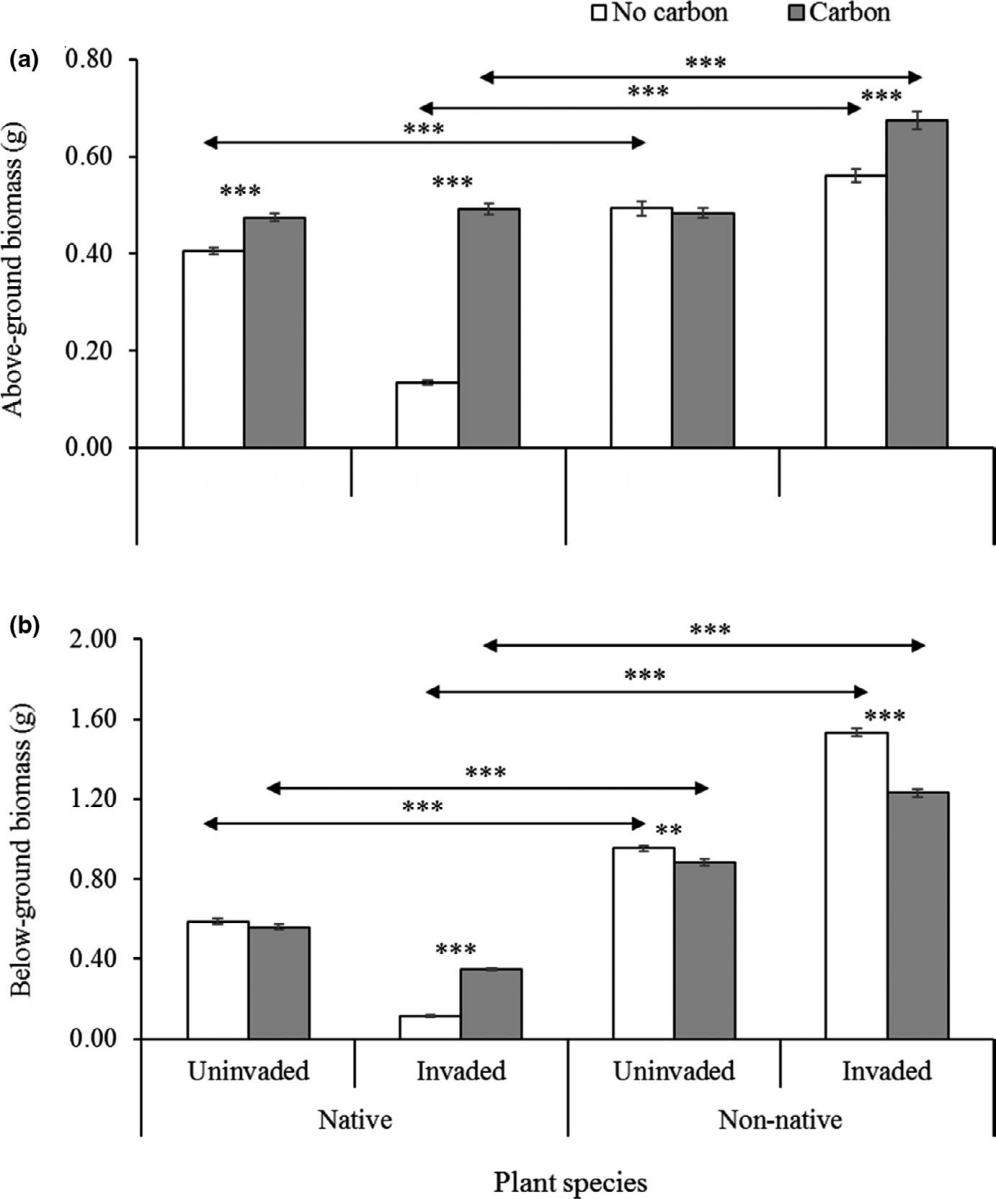
(a) Aboveground biomass and (b) belowground biomass of native *Juncus pallidus* and non‐native *Lolium rigidum* grass species grown in uninvaded and invaded rhizosphere soil of *Cynara cardunculus* either with or without activated carbon (AC). Each bar is the mean ± *SE*, *n = 21*. Asterisks above bar and horizontal line designate significant difference between AC treatments and target species, respectively, after independent sample *t* test (*p ≤ .05; **p ≤ .01 & ***p ≤ .001)

**FIGURE 5:**
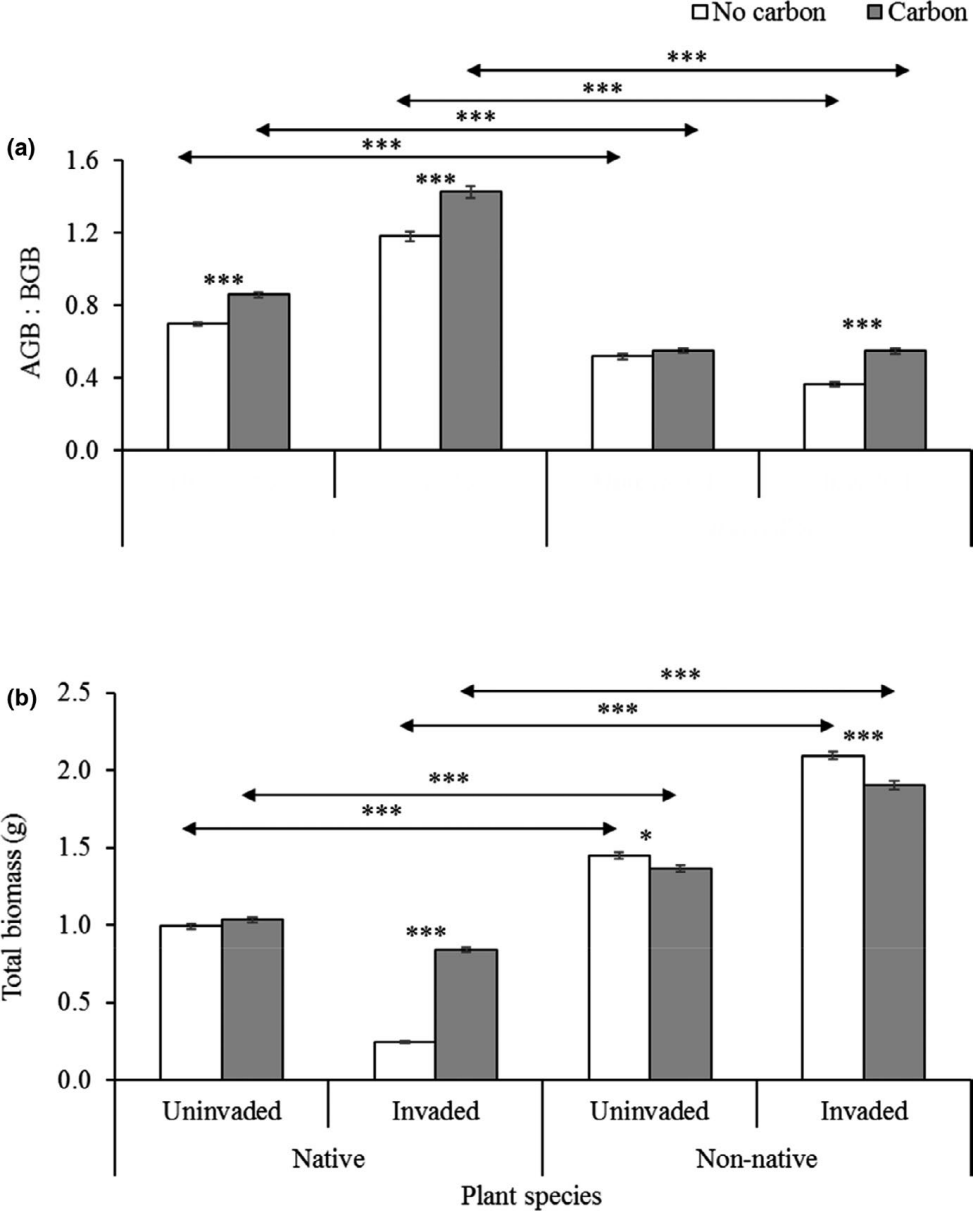
(a) Aboveground biomass (AGB): belowground biomass (BGB) and (b) total biomass of native *Juncus pallidus* and non‐native *Lolium rigidum* grass species grown in uninvaded and invaded rhizosphere soil of *Cynara cardunculus* either with or without activated carbon (AC). Each bar is the mean ± *SE*, *n = 21*. Asterisks above bar and horizontal line designate significant difference between AC treatments and target species, respectively, after independent sample *t* test (*p ≤ .05 & ***p ≤ .001)

The main and interactive effects of invasion status and carbon treatments on the growth performance of native and non‐native seedlings were presented in Table 1. The AC treatments in invaded rhizosphere soil had significant incremental effect on AGB, BGB, total biomass, AGB‐BGB ratio, plant height, and root length of native seedlings, whereas AGB and AGB‐BGB ratio of non‐native seedlings were positively affected (Table 1, Figures 3, 4, 5). In the invaded rhizosphere soil, AC significantly increased AGB (267%), BGB (202%), total biomass (237%), AGB‐BGB ratio (20.75%), maximum plant height (63%), and maximum root length (13%) for native seedlings, whereas the increased rate for non‐native was for AGB (20%) and AGB‐BGB ratio (49.59%) (Figures 3, 4, 5). Overall, AC in invaded soil had incremental effects on all measured biometric parameters of native seedlings, whereas most of the parameters except AGB, AGB‐BGB ratio, and maximum root length for non‐native seedlings were negatively affected (Figures 3, 4, 5). In uninvaded soil, AC treatments had significant incremental effects on AGB (17%), AGB‐BGB ratio (23.21%), and maximum root length (9.0%) for native seedlings, whereas only maximum root length (9.5%) of non‐native seedlings was positively affected (Figures 3, 4, 5). In most cases, AC in uninvaded soil had less significant effects on seedling performance of both species. On average, invaded rhizosphere soil of *C*. *cardunculus* showed greater negative effects on native seedlings compared with non‐native. The direct comparisons between native and non‐native grass species for measured parameters varied across treatments of invasion status (invaded and noninvaded) and AC (without and with AC; Figures 3, 4, 5).

**Table 1:** ANOVA of the effects of activated carbon (without carbon vs. with carbon), and plant invasion (uninvaded vs. invaded) on maximum plant height, maximum root length, aboveground biomass (AGB), belowground biomass (BGB), and total biomass of test plant species (native *Juncus pallidus* and non‐native *Lolium rigidum*)

Source of variation	*df*1, *df*2	AGB		BGB		AGB‐BGB ratio		Total biomass		Plant height		Root length	
		*F*	*p*	*F*	*p*	*F*	*p*	*F*	*p*	*F*	*p*	*F*	*p*
Native													
Activated carbon (AC)	1, 80	903.62	<.001	224.91	<.001	40.06	<.001	765.45	<.001	40.57	<.001	23.01	<.001
Plant invasion (PI)	1, 80	404.08	<.001	1,413.13	<.001	266.72	<.001	1,418.63	<.001	64.10	<.001	49.16	<.001
AC × PI	1, 80	472.75	<.001	299.58	<.001	0.32	.57	625.55	<.001	78.51	<.001	0.14	.710
Non‐native													
Activated carbon (AC)	1, 80	12.36	<.01	108.05	<.001	63.98	<.001	29.17	<.001	3.43	.067	5.72	<.05
Plant invasion (PI)	1, 80	85.80	<.001	667.07	<.001	33.71	<.001	563.17	<.001	1.63	.205	10.29	<.01
AC × PI	1, 80	17.77	<.001	42.55	<.001	32.88	<.001	4.92	.029	0.69	.408	1.14	.288

Note

Here, degrees of freedom 1 (*df*1) and degrees of freedom 2 (*df*2), where *df*2 equals the total number of observations in all cells (*n*) minus the degrees of freedoms lost because the cell means are set.

The direct effects of AC on experimental seedlings showed that there was no significant effect on the native seedlings in terms of AGB, BGB, total biomass, maximum plant height and root length and non‐native seedlings in terms of aboveground biomass, and maximum plant height and root length (all *p* > .05) except belowground biomass (*t* = 2.32, *df* = 8, *p* = .048). Actual data are provided in the online Appendix S1 (see Data S1).

## 4 DISCUSSION

### 4.1 Chemical changes in rhizosphere

Our results showed significant differences in phenolics content and pH level between rhizosphere soil of uninvaded and invaded plots in all populations. Many of the previous studies found inconsistent results for soil properties invaded by invasive plants under field conditions (Broz, Manter, & Vivanco, 2007; Ehrenfeld, 2003), but we followed the systematic sampling protocol to avoid the inconsistency in results. The findings of our studies aligned with other studies of *C*. *cardunculus*, those documented the high concentration of phenolic compounds in different organs of the plant (Ciancolini et al., 2013; Dias et al., 2018; Pandino, Lombardo, Mauromicale, & Williamson, 2011a, 2011b). The high concentration of allelochemicals in plant organs has led to the suggestion that soil may receive a major portion of the released chemicals from plants through leaching, rhizodeposition, litter decomposition, etc. and might be involved in allelopathic effects between neighboring plant species (Kong, Xuan, Khanh, Tran, & Trung, 2019; Kong et al., 2018). Furthermore, the results were supported by other studies, which found high phenolic concentration in soil invaded by invasive plants such as *Pluchea lanceolata*, *C. stoebe, Mikania micrantha*, and *Phragmites australis* (Bais et al., 2003; Hierro & Callaway, 2003; Inderjit, 1998; Kaur, Malhotra, & Inderjit, 2012; Rudrappa, Bonsall, Gallagher, Seliskar, & Bais, 2007; Nazim & Robinson, 2017a, 2017b).

In addition to the increased phenolic content in the invaded soil of *C*. *cardunculus*, our studies found lower pH level, which seems to be associated with the phenolic compounds in the plants that might accumulate in rhizosphere through root exudates, leachate, and litter decomposition. The invasive plants including *Solidago gigantea* (Herr, Chapuis‐Lardy, Dassonville, Vanderhoeven, & Meerts, 2007; Scharfy, Eggenschwiler, Venterink, Edwards, & Gusewell, 2009), *Phragmites australis* (Nazim & Robinson, 2017a, 2017b), *Eucalyptus camaldulensis* (Soumare et al., 2016), and *Rudbeckia laciniata* (Stefanowicz, Majewska, Stanek, Nobis, & Zubek, 2018) lowered the pH level in the invaded soil, which support our findings. On the contrary, some studies regarding invasive plants like *Alliaria petiolata* (Rodgers, Wolfe, Werden, & Finzi, 2008), *Mikania micrantha* (Kaur et al., 2012), and *Kalmia angustifolia* (Inderjit & Mallik, 1999) increased the pH level in the invaded soil.

### 4.2 Germination and growth performance

Our results found *C*. *cardunculus* invaded soil had a significant negative effect on the germination percentage and growth parameters of native, but less significant effect on non‐native co‐occurring grass species, indicating that the result depended intricately on the identity of test species. The total effect of allelopathy was greater on the native grass species, as the germination and total biomass increased more significantly in the invaded soil manipulated with AC. The AC neutralized the negative effects of allelochemicals available in soil, whereas the non‐native grass species showed almost no sensitivity to allelochemicals. Goslee, Peters, and Beck (2001) found that moderate sensitivity to allelochemicals induced by *Acroptilon repens* was an important component to swing the consequences among neighboring species competition in grasslands.

The approach in our study using invaded rhizosphere soil is more ecologically realistic than general bioassay because the use of plant extracts lacks the ability to quantify or identify the response of allelochemicals available in invaded soil system, and whether the concentration of allelochemicals is responsible for allelopathy. Our study did not cover the whole criteria of allelopathy research design documented by Williamson (1990) and Blum, Shafer, & Lehman (Blum, Shafer, & Lehman, 1999) to demonstrate allelopathy of *C*. *cardunculus*. However, we used AC in field‐collected soil to separate the allelopathic effects on co‐occurring plant species from other possible effects that could demonstrate the allelopathy in a more ecologically realistic way. This approach may provide a better understanding of allelopathy under soil chemical ecological context. The previous studies of *C*. *cardunculus* demonstrated phytotoxicity of sesquiterpene lactones as secondary metabolites derived from different parts of the plant (Rial et al., 2014, 2016), but our studies did not isolate the individual chemical from the invaded soil. Isolation of those chemicals from rhizosphere, and the implications of those under settings that are more natural is required, to evaluate its extent compared to resource competition or other interactions (Scavo, Abbate, et al., 2019).

Our results found native species *J. pallidus* were more sensitive to allelochemicals available in rhizosphere of *C*. *cardunculus*, whereas non‐native *L. rigidum* showed less sensitivity. The AC increased more significantly of germination percentage, growth parameters namely AGB, BGB, total biomass, and maximum plant height and root length of native grass species, whereas only AGB increased for non‐native. The effect of AC may provide the evidence of allelopathy (Adomako et al., 2019; Callaway & Aschehoug, 2000; Lyytinen & Lindstrom, 2019; Mahall & Callaway, 1991), but there is debate to use AC for allelopathy test, as it may also influence other soil properties such as nutrient availability (Lau et al., 2008), and biological properties, namely microbial communities in soil (Weißhuhn & Prati, 2009). Nevertheless, the results in our separate experiment showed no significant direct effect of AC on the measured biometric parameters including AGB, and total biomass of any of the two grass species.

The non‐native invasive plant species might achieve success in their introduced range due to allelopathy this being the alternative mechanism for their “evolution of increased competitive ability” (Hierro & Callaway, 2003; Zheng et al., 2015). This hypothesis explains invasive plants achieve greater competitive advantages over resident species due to lack of coevolved tolerance to new allelochemicals compared to their original ranges, referred to as “allelopathic advantage against resident species” hypothesis proposed by Callaway and Ridenour (2004; Inderjit, Callaway, & Vivanco, 2006). Thus, it is evident that native species experience greater effects through induced allelochemicals by invasive species compared to non‐natives, which had same original range of the invaders. In our studies, the tested non‐native grass species *L. rigidum* co‐occur with *C. cardunculus* in their native range (Müller, Deil, De Mera, & Orellana, 2005), which may provide a comparative evidence of coevolved tolerance to allelochemicals induced by *C. cardunculus*.

Our studies found native *J. pallidus* experienced strong allelopathic effects, whereas non‐native *L. rigidum* showed more resistance to allelochemicals indicating that *L. rigidum* is less susceptible to allelochemicals induced by *C. cardunculus*. This may explain the coevolutionary history of the *L. rigidum* with *C. cardunculus* in its native range (i.e., southern Europe), which may provide more competitive advantages over native species. It is evident that the species *L. rigidum* coevolves with *C*. *cardunculus* over a longer period in their native ranges that may provide resistance to allelochemicals induced by *C*. *cardunculus*. The findings are more aligned with the studies of Hu and Zhang (2013) and Prati and Bossdorf (2004) whom found, for example, *Alliaria petiolata* had greater allelopathic effects on native (American) *Geum laciniatum* germination, but less effect on non‐native (European) *Geum urbanum*. In addition, *Chromolaena odorata* showed significant allelopathic effects on five native species (China) compared to non‐native (South American) species. In contrast, Mallik and Pellissier (2000) found that invasive *Vaccinium myrtillis* had significant allelopathic effects on the non‐native *Picea mariana* in comparison with the native *Picea abies*. This indicates plant species without a common evolutionary history exert significant allelopathic interactions. This is also supported with the notion of a coevolutionary aspect to allelopathy (Arroyo, Pueyo, Saiz, & Alados, 2015; Rabotnov, 1981; Thorpe, Thelen, Diaconu, & Callaway, 2009).

### 4.3 Linking changes in soil properties and plant communities

Invasive plant species may influence rhizosphere properties rapidly from surrounding native communities, which have major impact on the plant communities (Gibbons et al., 2017; Weidenhamer & Callaway, 2010). The plant‐driven changes in physical, chemical, or biological soil properties may significantly influence germination and growth performance of the neighboring plant species (Chen, Wang, Wang, & Kong, 2014; Gentili, Ambrosini, Montagnani, Caronni, & Citterio, 2018; Herranz, Ferrandis, Copete, Duro, & Zalacaín, 2006; Lyytinen & Lindstrom, 2019; Miller, Perron, & Collins, 2019). The chemical soil characteristics influenced by allelochemicals of invader might play an important role in ecological implications between plants and soil systems (Scavo, Abbate, et al., 2019). Among all the changes, the allelopathic attributes of some invasive plants make them more aggressive. Allelopathy as an invasion mechanism of some invaders in natural plant communities remains a disputed ecological research matter, partly due to lack of evidence of allelochemicals persistence and effects in the surrounding plants. The establishments of the allelopathic interactions need the demonstration of allelopathy in invaded rhizosphere under soil chemical ecological context (Blum et al., 1999; Scavo, Abbate, et al., 2019). Thus, our studies demonstrated that the chemical changes in rhizosphere induced by *C. cardunculus*. had significant negative allelopathic effects on co‐occurring native grass species compared to non‐native. This also leads to explain that the non‐native grass species might have a long coevolution history with *C. cardunculus*, and thus, the evolution influenced the allelopathic interactions among them.

Our studies may contribute to the existing body of evidence demonstrating that changes in soil properties induced by invasive plants like *C. cardunculus* may influence the composition of the plant communities. The plant‐driven changes in soil properties and thereby the altered functions and processes might create feedback mechanisms, which may proliferate the invasibility of the invader (Ehrenfeld & Scott, 2001; Inderjit & Cahill, 2015; Weidenhamer & Callaway, 2010). This is of a particular concern because such mechanisms might have significant implications for management of invasions and restoration of native communities in the invaded communities.

## 5 CONCLUSIONS

In summary, the results of our studies recommend that allelopathy of *C*. *cardunculus* can contribute to its success through negative impacts on the natives as an invasive species in Australian agricultural fields. The allelopathic interference was species‐specific as a function of coevolutionary context, which is mostly imperative for invasive species, as the invaders compete with diverse sets of species (native and non‐native) in different ranges. We recommend further works to evaluate the relative allelopathic effects by *C*. *cardunculus* on co‐occurring species of “origin” versus *“*recipient” plant communities, considering the concentration of natural allelochemicals in soil that may demonstrate the generality of allelopathy to the better understanding of plant invasions. Furthermore, studies regarding species‐specific rhizosphere allelochemistry evaluating evolved tolerance of test species may be recommended for ecologically realistic and meaningful information.

## CONFLICT OF INTEREST

Authors declare no conflict of interest.

## AUTHOR CONTRIBUTION

**Md Nazim Uddin:** Conceptualization (lead); Data curation (lead); Formal analysis (lead); Funding acquisition (lead); Investigation (lead); Methodology (lead); Project administration (lead); Resources (equal); Software (lead); Supervision (lead); Validation (lead); Visualization (lead); Writing‐original draft (lead); Writing‐review & editing (lead). **Takashi Asaeda:** Conceptualization (supporting); Funding acquisition (supporting); Investigation (supporting); Project administration (supporting); Supervision (equal); Validation (equal); Writing‐original draft (supporting); Writing‐review & editing (supporting). **Shahana Haque Shampa:** Conceptualization (supporting); Data curation (equal); Investigation (supporting); Methodology (supporting); Project administration (supporting); Writing‐original draft (supporting); Writing‐review & editing (supporting). **Randall Robinson:** Conceptualization (equal); Investigation (equal); Methodology (equal); Project administration (equal); Resources (equal); Supervision (equal); Validation (equal); Writing‐original draft (supporting); Writing‐review & editing (equal).

## Supporting information

AppendixS1

## Data Availability

Uddin, Md. Nazim; Asaeda, Takashi; Shampa, Shahana H.; Robinson, Randall W. (2020), Allelopathy and its coevolutionary implications between native and non‐native neighbors of invasive Cynara cardunculus L., Dryad, Dataset, https://doi.org/10.5061/dryad.f7m0cfxsg
