# Supplementary material for: Allelopathy and its coevolutionary implications between native and non‐native neighbors of invasive Cynara cardunculus L
Source: Ecol Evol. 2020 Jun 28;10(14):7463–75. doi: 10.1002/ece3.6472 (PMC7391558; doi:10.1002/ece3.6472)
Supplement: Supplementary file 1 — AppendixS1 [file ECE3-10-7463-s001.docx]

**Appendix S1**. Direct effects of activated carbon (AC) on seedlings of native *Juncus pallidus* and non-native *Lolium rigidum* grass species used in the experiments. Values were expressed as mean ± *SE*, *n = 5*. Similar letters indicate that treatments do not differ significantly at *P* ≤ 0.05 after independent sample *t*-test.

| Plant materials | Without AC | With AC |
| --- | --- | --- |
| *Native* |  |  |
| Above ground biomass (g) | 0.46 ± 0.017^a^ | 0.51 ± 0.019^a^ |
| Belowground biomass (g) | 0.60 ± 0.036^a^ | 0.64 ± 0.029^a^ |
| Total biomass (g) | 1.06 ± 0.051^a^ | 1.15 ± 0.042^a^ |
| Maximum plant height (cm) | 27.40 ± 1.029^a^ | 29.60 ± 1.720^a^ |
| Maximum root length (cm) | 21.80 ± 1.157^a^ | 22.60 ± 1.208^a^ |
| *Non-native* |  |  |
| Above ground biomass (g) | 0.556 ± 0.020^a^ | 0.554 ± 0.016^a^ |
| Belowground biomass (g) | 1.026 ± 0.028^a^ | 0.938 ± 0.024^b^ |
| Total biomass (g) | 1.582 ± 0.031^a^ | 1.492 ± 0.054^a^ |
| Maximum plant height (cm) | 30.80 ± 1.356^a^ | 27.4 ± 0.812^a^ |
| Maximum root length (cm) | 17.00 ± 0.707^a^ | 16.00 ± 1.224^a^ |
